# T Cell-Mediated Immune Responses to AAV and AAV Vectors

**DOI:** 10.3389/fimmu.2021.666666

**Published:** 2021-04-13

**Authors:** Hildegund C. J. Ertl

**Affiliations:** Wistar Institute, Philadelphia, PA, United States

**Keywords:** CD8^+^ T cells, memory, immunosuppression, effector functions, animal models, clinical trials

## Abstract

Adeno-associated virus (AAV)-mediated gene transfer has benefited patients with inherited diseases, such as hemophilia B, by achieving long-term expression of the therapeutic transgene. Nevertheless, challenges remain due to rejection of AAV-transduced cells, which in some, but not all, patients can be prevented by immunosuppression. It is assumed that CD8^+^ T cells induced by natural infections with AAVs are recalled by the AAV vector’s capsid and upon activation eliminate cells expressing the degraded capsid antigens. Alternatively, it is feasible that AAV vectors, especially if given at high doses, induce *de novo* capsid- or transgene product-specific T cell responses. This chapter discusses CD8^+^ T cell responses to AAV infections and AAV gene transfer and avenues to prevent their activation or block their effector functions.

## Introduction

The goal of gene therapy is to permanently replace a missing or faulty gene and thereby through sustained production of the transgene product achieve a functional cure. Various methods have been explored to insert genes *in situ* into specific cells (1, 2). One of the most promising gene transfer vectors are AAV vectors, which in initial preclinical studies achieved sustained expression of their transgene product in mice (3), dogs (4), and nonhuman primates (5) without any overt serious adverse events. In humans clinical trials targeting Leber’s congenital amaurosis, a congenital form of blindness, by small doses of AAV injected into the subretinal space reported long-term improvement of vision (6, 7). In contrast, the first clinical trial for hepatic AAV-mediated transfer of factor (F)IX for correction of hemophilia B accomplished initial increases in F.IX levels, which were followed a few weeks later by a subclinical transaminitis and loss of F.IX (8). Additional studies showed that patients developed concomitantly with rises in liver enzymes circulating CD8^+^ T cells to AAV capsid antigens (9). This led to the still valid but nevertheless unproven hypothesis that patients had AAV-capsid-specific memory CD8^+^ T cells, which were reactivated by the gene transfer and then eliminated the vector-transduced hepatocytes (10).

This opened a slurry of pre-clinical experiments that aimed to recapitulate the findings of the clinical trial. Although the animal experiments allowed the field to gain valuable knowledge of the intricacies of anti-AAV capsid T and B cell responses (11–13), in the end the studies confirmed what we have known for long – mice are not humans (14) and neither mice nor larger animals are overly informative about the presumably immune-mediated rejection of AAV-transduced cells.

Clinical AAV-mediated gene transfer trials by reducing vector doses and using various immunosuppressive regimens at least in part overcame immunological barriers and achieved treatment benefits or even cures for their patients (15, 16). Nevertheless, transfer of genes with high doses of AAV remains a crapshoot especially in 2020/21 during a global pandemic with a potentially fatal virus that is especially dangerous for immunocompromised humans (17). Immune responses to AAV gene transfer are complex involving both the innate and adaptive immune systems. Here we discuss what is known from pre-clinical models as well as clinical trials about CD8^+^ T cells to AAV gene transfer.

## AAV Virus and Immune Responses to Natural Infections

AAVs are single-stranded DNA viruses of the parvovirus family. As dependoviruses they only replicate in presence of a helper virus such as an adenovirus. AAVs do not cause any known disease. The ~4,700 base pair long AAV genome, which is flanked by inverse terminal repeats (ITRs), has two open reading frames, one for rep proteins needed for viral replication, and the other for the capsid proteins vp1, vp2 and vp3, which are produced by differential splicing and therefore only differ in their N-terminus (18). Capsid proteins distinguish serotypes of AAV. Thus far 12 human serotypes of AAV have been identified (19). They differ in their tropism (20) and in the prevalence, with which they circulate in humans (21). AAV genomes persist mainly episomally in the nucleus of infected cells although they can integrate into a specific site of human chromosome 19 (22).

Humans, who become naturally infected with AAVs, mount adaptive immune responses, which presumably are in part driven by innate responses to the helper virus (23). Prevalence rates of neutralizing antibodies to different serotypes of AAVs, which serve as indicators for previous infections, vary in part depending on age and country of residency (21, 24–31). Some studies report strikingly different prevalence rates even when they tested similar populations. This likely reflects that AAV neutralization assays are not standardized and therefore differ in their sensitivity. Overall trends are similar. Prevalence rates of neutralizing antibodies to AAV increase with age and they are higher for AAV2 or AAV8 than for example AAV5 or AAV6.

T cell responses have been studied less well. We reported that about 50% of healthy human adults have detectable frequencies of circulating AAV capsid-specific CD8^+^ and/or CD4^+^ T cells when tested by intracellular cytokine staining (ICS); 50% of these CD8^+^ T cells belong to the central memory subsets and 25% each to the effector and effector memory subsets. AAV capsid-specific CD4^+^ T cells belong mainly to the central memory subset (32). Non-human primates tested by the same method showed that 5 out of 6 have AAV capsid-specific CD8^+^ T cells while 6/6 have CD4^+^ T cells of that specificity. In monkeys, CD8^+^ T cells are strongly biased towards effector cells (32). For these assays we used a peptide panel that reflected the capsid sequence of AAV2 but would like to point out that many of the T cell epitopes are highly conserved. Nevertheless, unlike in humans AAV-mediated gene transfer achieves long-lasting transgene product expression in nonhuman primates, which may reflect that their T cells potentially due to high levels of persisting AAVs are functionally exhausted (32). Overall, not only prevalence but also frequencies of AAV capsid-specific T cells are higher in non-human primates than in humans. Testing additional non-human primates by an ELISPOT assay, which is the assay that is primarily being used by gene therapists to monitor T cell responses to AAV capsid upon AAV-mediated gene transfer, showed lower prevalence rates of AAV capsid-specific T cells of ~50% (32).

Using a proliferation and cytokine secretion assays another group reported that peripheral blood mononuclear cells (PBMCs) of less than 10% of humans mount a response (29) although it should be pointed out that these assays lack sensitivity. Another group using ELISPOT assays as well as ICS showed with either assay that ~30% of health human adults respond to AAV1 capsid (33). A study using a very sensitive method based on pre-selection of AAV8-specific CD8^+^ T cells with a specific tetramer showed that all tested humans have circulating effector memory CD8^+^ T cells against AAV8 capsid (34). Human circulating AAV capsid-specific CD8^+^ T cells are functional, they secrete cytokines (32, 34) and lyse target cells expressing their cognate antigen (33). T cell epitopes are conserved between several AAV serotypes (9) and several studies reported no correlations between antibody and CD8^+^ T cell responses (32, 35). One study showed that peripheral blood mononuclear cells from AAV2 seronegative donors mount a robust IFN-g-secreting natural killer cell response to *in vitro* culture with an AAV capsid peptides while those from seropositive individuals showed activation of tumor necrosis factor-α producing CD8^+^ T cells (36).

Overall, these data demonstrate that AAV infections are highly prevalent and cause sustained immunological memory that can presumably be recalled upon re-infection or transfer of an AAV vector.

## AAV Vectors

Production and purification methods for AAV vectors are well established (37). In AAV vectors the viral genes but for the inverted terminal repeats (ITRs) are replaced with an expression cassette for a therapeutic protein. A variety of promoters have been used, some of which drive ubiquitous expression while others are specific for selected cell types. For some applications, such as hemophilia B, a variant transgene with improved functions compared to protein encoded by the wild-type gene has been used to allow for dose-sparing.

AAV vectors are in general generated by triple transfection of a cell line, such as HEK 293 cells, which carry the E1 gene of adenovirus. One plasmid expresses additional adenoviral genes to promote AAV production. A second plasmid carries the AAV cap and rep genes. The AAV2 rep gene is used for most AAV vectors while the cap gene determines the serotype of the vector. The third plasmid carries the transgene expression cassette flanked by the ITRs, again most commonly of AAV2. Vectors are then released from the transfected cells and purified by various methods such as gradient centrifugation, column purification and others (38). The type of purification may affect levels of empty AAV particles within the preparation, which in turn can influence the induction of immune responses or reduce the inhibitory effects of AAV neutralizing antibodies.

AAV vectors can also be produced in the baculovirus expression system, which is more amenable for scale-up than mammalian expression systems (39). AAVs produced in mammalian cells or insect cells show differences in post-translational modification, genome methylation and levels and types of host cell contaminations which affect their immunogenicity and their performance in clinical trials (40, 41).

## CD8^+^ T Cells

CD8^+^ T cells are uniquely capable to eliminate virus-infected or vector-transduced cells by direct lysis mediated by the release of perforin and granzyme. They also secrete anti-viral cytokines such as interferon (IFN)-γ. Activation of naïve CD8^+^ T cells, which reside in lymphatic tissues and circulate in blood, requires presentation of antigen-derived peptides bound to major histocompatibility complex (MHC) class I molecules by professional mature antigen-presenting cells. Peptides can be generated from *de novo* synthesized misfolded proteins that upon degradation by proteasomes are transported by the transporter associated with antigen presentation (TAP) into the endoplasmic reticulum (ER), where they bind to MHC class I molecules, which are then transported to the cell surface. This classical presentation pathway would apply to vector-encoded transgene products. Antigen-presenting cells are able to cross-present protein such as those of the AAV capsid that are taken up by pinocytosis, phagocytosis or endocytosis. In the so-called cytosolic pathway, the particles are degraded in phagosomes and peptides are released into the cytoplasm from where they can be transported into the ER; there they can bind to MHC molecules (42). In the vacuolic pathway, proteins are degraded within endosomes. They escape into the cytoplasm upon acidification of the endosomes or upon reactive oxygen species-mediated lipid peroxidation of endosomal membranes (43).

The antigenic peptides displayed by MHC class I antigens on the cell surface bind to T cell receptors, which triggers a signaling cascade that through the adaptor molecule zeta-chain-associated protein kinase 70 (ZAP70) induces activation of calcineurin leading to the activation of nuclear factor of activated T-cells (NFAT). Full activation of T cells furthermore requires interactions with co-stimulators most commonly CD80 and CD86 or CD40 on antigen-presenting cells, which interact with CD28 or CD40L on T cells, respectively. This amplifies T cell receptor signaling and through phosphoinositide 3-kinase (PI3K) induces the mechanistic target of rapamycin (mTOR)/protein kinase B (Akt) pathway which modifies the T cells’ metabolism to provide energy and building blocks for rapid proliferation. Dendritic cells, the main cell type that presents antigens to naïve T cells, are immature when they are released from bone marrow. At this stage they do not express co-stimulators and are therefore unable to activate an effector CD8^+^ T cell response but rather induce tolerance. Maturation of dendritic cells into profession antigen-presenting cells is driven by pathogen-associated molecular patterns (PAMPs), such as CpG motifs within the genome, which are common in bacteria and viruses but largely absent in mammalian cells. PAMPs interact with pathogen recognition receptors (PRR) such as Toll-like receptors (TLRs) and others expressed in different cellular compartments (44). Binding of a PAMP to a PRR causes activation of numerous pathways, such as the nuclear factor kappa B (NK-κB) and interferon regulatory factor (IRF)3 pathways. Induction of these pathways, which can also be activated by type I interferons (IFN) or members of the tumor necrosis factor (TNF) family, involves a number of molecules such as TIR domain containing adaptor protein (TIRAP), myeloid differentiation primary response 88 (MyD88), inhibitor of NF-κB kinase (IKK)-γ, or interleukin-1 receptor-associated kinase (IRAK)-4, all of which can be targeted by drugs to block inflammatory responses. Once NF-κB or IRF-3 are activated they induce pro-inflammatory cytokine responses, which initiate or increase production of molecules that are essential for antigen processing and presentation. Upon stimulation, CD8^+^ T cell proliferate very rapidly and then migrate to sites of infection where they assume effector functions. Recognition of foreign antigen is exquisitely sensitive and can be triggered by as few as 2-3 MHC-peptide complexes on the surface of a cell (45). CD8^+^ T cell differentiation requires help from CD4^+^ T cells (46) belonging to the T helper (Th)1 subset. Once the antigen has been removed most of the effector CD8^+^ T cells die, some will differentiate into memory cells, which can be recalled rapidly. Re-activation of memory CD8^+^ T cells does not require professional antigen presenting cells and is less dependent on co-stimulation. Effector CD8^+^ T cells can differentiate into different type of memory cells, i.e., effector, central memory or tissue resident memory T cells. Effector memory CD8^+^ T cells circulate. They do not proliferate extensively after re-exposure to antigen and can assume functions instantly. Over time in absence of antigen they differentiate into central memory cells or die. Central memory CD8^+^ T cells reside in lymphatic tissues. They do not exhibit functions. Upon reencounter of their antigen they proliferate vigorously before they assume effector functions; this may take several days. Central memory CD8^+^ T cells are maintained at steady numbers potentially throughout the lifespan of an individual. Tissue resident memory CD8^+^ T cells are also very long-lived but they remain at sites of previous infections. Upon local reinfection they can immediately release cytolytic enzymes and initiate an inflammatory reaction. If the antigen is not removed but continues to persist at high levels, T cells will differentiate towards exhaustion by gradually losing function, increasing expression of co-inhibitors such as programmed cell death protein 1(PD-1) and eventually undergoing apoptosis (47).

How does this apply to AAV vectors? To activate naïve CD8^+^ T cells one would expect that the vector would be phagocytosed by immature dendritic cells. PAMPs within the vector genome or on the capsid would interact with PRRs on or within the cells. This would trigger an inflammatory reaction and maturation of the dendritic cells, which would then migrate to draining lymph nodes. Within cells antigens encoded by the AAV vector would enter the classical presentation pathway while antigens of the AAV capsid would be processed and presented by either the cytosolic or vacuolic pathway. Dendritic cells presenting antigen bound to MHC class II molecules would activate specific CD4^+^ T cells which would then facilitate stimulation followed by expansion of CD8^+^ T cells by antigen displayed by MHC class I antigen. Subsequently T cells would migrate out of lymph nodes and circulate till they find their cognate antigen. Cells displaying this antigen would be killed rapidly within minutes and then the T cell would find its next target. This process would continue till all of the antigen is removed, which in case of AAV particles may take months. Memory CD8^+^ T cells generated in response to a natural infection can be stimulated by cells other than dendritic cells and depending on subset they can act immediately once they see their antigen displayed on MHC class I. If they belong to the tissue resident memory CD8^+^ T cell subset they may not even proliferate, which raises the question if screening for increases in circulating T cells is adequate to predict immune-mediated rejection of AAV-transduced cells. Due to higher numbers of precursors memory T cell responses are more potent and come up more rapidly.

It is assumed that the increases of circulating AAV capsid specific CD8^+^ T cells reflect recall of memory cells that had initially been activated by a natural infection. This may be the case for some patients but activation of naïve CD8^+^ T cells should not be ruled out. The very slow increases of AAV capsid-specific T cells in some AAV vector recipients would be more typical for primary than secondary responses. As stimulation requirements and thereby sensitivity to immunosuppressive drugs differ for naïve and memory CD8^+^ T cells further studies are needed to elucidate what T cell subsets respond to AAV gene transfer.

## CD8^+^ T Cell Responses to AAV Vectors

Viral vectors can induce CD8^+^ T cell responses to their own antigens as well as to a transgene product. In the case of AAV vectors, which have been stripped of genes that encode AAV proteins, any effector T cell response to the viral proteins would be limited to the time frame till all the vector particles have been completely degraded. T cell responses to the transgene product on the other hand could continue till all antigen-producing cells have been removed or till immunosuppressive mechanisms such as T cell exhaustion or regulatory T cells turn off the T cells.

Initial studies reported that AAV vectors did not induced CD8^+^ T cell responses to the transgene product and this was attributed to lack of activation of innate responses, which resulted in immunological ignorance (48). Additional studies contradicted these results and reported that AAV vectors can induce transgene product-specific CD8^+^ T cell responses in experimental animals (49–51). It was also shown that AAV vectors elicit albeit weak and transient innate responses that are largely driven by TLR9 activation through CpG motifs within the vector genome (52) or TLR2 activation by capsid components (53). Naturally immunogenic transgene products, such as antigens from another pathogens, induce depending on the vector’s serotype and it’s genome structure such robust immune responses that AAV vectors were explored as vaccine carriers (50, 51). The magnitude of the immune responses depends on vector dose, the AAV serotype, the transgene, the type of promoter, the target tissue and the vectors’ genome structure (54). Self-antigens, such as F. IX with point mutations, are non-immunogenic while the same mutant F.IX in a mouse with a genetic F.IX deletion induces cellular and humeral responses (55). Comparing vectors with single-stranded and double-stranded DNA genomes, showed that the latter are more immunogenic presumably by inducing more potent innate responses (56). Some studies showed that CD8^+^ T cell responses induced by an AAV vector-encoded transgene are defective in mice: T cells do not proliferate upon re-exposure to their antigen *in vivo*, they only produce low levels of cytokines and they fail to protect against a surrogate pathogen (57, 58). Others showed that the effectiveness of transgene product-specific hepatic CD8^+^ T cell responses is dependent on vector dose; intermediate doses of vector lead to a delayed CD8^+^ T cell response that eliminates antigen-producing hepatocytes. High doses of vector induce multiple immunosuppressive pathways that block induction of transgene product-specific CD8^+^ T cells (59).

AAV vectors can induce capsid-specific CD8^+^ T cell responses, especially if highly immunogenic T cell epitopes are incorporated into the capsid (60, 61). This process is likely driven by cross-presentation of capsid proteins and not only requires plasmacytoid and conventional dendritic cells but also help from CD4^+^ T cells (62, 63). Nevertheless, in mice specific CD8^+^ T cells induced by AAV gene transfer fail to eliminate AAV-transduced cells (64), which can be achieved by adaptive transfer of *ex vivo* expanded capsid-specific CD8^+^ T cells suggesting defects at the level of T cell differentiation *in vivo* that could be overcome in tissue culture (65).

Although they fail to reject AAV-transduced cells, mice have been useful to study the duration of capsid degradation, which in the end dictates how long gene transfer recipients are at risk to lose treatment benefit due to AAV capsid-specific CD8^+^ T cells. Experiments using proliferation of capsid-specific CD8^+^ T cells, which were adoptively transferred into AAV-injected mice as a read-out, showed that T cells proliferated in their hosts even if transferred 6 months after AAV injection, which is reflective of the very slow degradation of AAV capsid (66).

Clinical trials using different serotypes of AAV vectors over a large range of doses have been completed, are ongoing or planned for a number of diseases. For ocular diseases such as choroiderma, an X-linked form of progressive vision loss (67), achromatopsia or color blindness (68), X-linked retinitis pigmentosa (69), or Leber’s congenital amaurosis (70) AAV vectors encoding the therapeutic protein are injected at modest doses into the subretinal space, which similar to the central nervous system is an immunoprivileged site (71) that contains high levels of transforming growth factor (TGF)-ß and is shielded by a physical barrier from blood. In addition, parts of the eye, such as the ocular chamber, actively induce immune tolerance through a process called anterior chamber-associated immune deviation (ACAID)  (72). It should be noted though that this pathway does not induce systemic tolerance after subretinal injection of an AAV vector (73). While some trials for correction of ocular diseases reported stable transgene product expression for years without evidence for induction of T cell responses (74, 75), others observed stimulation of adaptive immune responses combined in some cases with loss of therapeutic benefits (76). Pre-clinical studies indicate that induction of immune responses to ocular injection of AAV vectors depends on vector dose, the promoter, route of application and the transgene (77), which may in part explain discrepancies of results.

Many AAV-mediated gene transfer trials focus on hemophilia where circulating coagulation factors offer an easy read-out for transgene product expression. Vectors that were or are being explored for hemophilia B include AAV8, AAV5, AAVrh10 or AAVs with genetically engineered capsids expressing either wild-type F.IX or the 5 time more potent F. IX Padua variant. Vectors either contain a single-stranded or self-complementary genome. They were or are given at doses ranging from 2x10^11^ to 2 x 10^12^ vg/kg achieving post-infusion levels of F.IX depending on the trial and the vector dose from ~1-40% of normal. Many trials use codon-optimized vectors to increase expression and/or vectors in which most CpG motifs are modified to minimize TLR9 activation. Some trials, such as one using AAV5 expressing the Padua variant of F.IX failed to observe any post-infusion transaminitis (78), which would be indicative of an anti-AAV immune response while others using the same vector reported transient increases in liver enzymes, which were not accompanied by detectible T cell responses (79). Using an AAV vector with a bioengineered capsid expressing F.IX Padua or a self-complementary AAV8 vector expressing wild-type F.IX other groups reported in some patients increases in transaminases combined with increases of circulating AAV capsid-specific T cells (80, 81). Data have been published for one hemophilia A trials, which used a baculovirus-derived AAV5 vector. The study reported increases in liver enzymes but failed to detect concomitant rises in capsid-specific circulating T cells (80). Circulating FVIII levels tended to decrease gradually by 2-3 years after gene transfer most likely reflecting hepatocyte turn-over rather than an immune-mediated rejection (16). A number of additional trials that are ongoing for correction of hemophilia A have reported therapeutic benefits (82) but not yet released potential problems with immune-mediated rejection.

Overall transfer to the liver, a unique microenvironment that favors immunosuppression (83), can induce in a dose-dependent manner capsid-specific CD8^+^ T cell responses, which have been implicated to eliminate AAV-transduced cells. Thus far transgene product-specific CD8^+^ T cells have not yet been observed upon hepatic transfer of AAV vectors. This may in part reflect that trials mainly enrolled patients with point mutations in their coagulation factors, which destroy their biological activity but fail to prevent induction of immunological tolerance. Most patients had also received factor replacement therapy, which would further promote tolerance. It would be expected that AAV vectors will induce F.VIII- or F.IX-specific T cell responses in patients with large deletion mutations although such responses might potentially be dampened or blocked by concomitant induction of regulatory T cells (59).

For treatment of other diseases AAV vectors are given into the muscle. For example, for treatment of Pompe disease, caused by glycogen storage in muscle and motor neurons due to lack of lysosomal alpha-glucosidase, an AAV9 vector was injected at a high dose into the diaphragm in children with progressive respiratory failure requiring ventilation and enzyme replacement therapy (84). T cell responses to the vector or transgene product were not detected. Several trials are exploring intramuscular injection of AAV vectors expressing dystrophin for treatment of Duchenne’s muscular dystrophy (85, 86) or a1-antitrypsin (AAT) deficiency (87, 88). An AAT trial using an AAV-1 vector reported that vector-derived AAT levels were sustained for several months although all treatment recipients developed T cell responses to the capsid proteins of AAV1 and 1 of the 9 patients had at one timepoint a positive T cell response to AAT. A follow-up study conducted about a year later showed sustained presence of the AAV genome in the injected muscle and a marked reduction in inflammatory cells in year 1 compared to months 3 biopsy samples. A substantial portion of the muscle infiltrating lymphocytes were regulatory T cells suggesting that they had suppressed vector-induced effector T cell responses and thereby prevented loss of AAV-transduced muscle cells (87, 88). A further follow-up study conducted 5 years after gene transfer detected Tregs and AAV capsid-specific CD8^+^ T cells within the injected muscle (89).

For AAV-mediated correction of Duchenne’s muscular dystrophy one trial reported that patients developed no or only very weak T cell responses to the viral capsid but instead some generated robust transgene product-specific CD8^+^ T cell responses (90, 91). AAV1-mediated transfer of the alpha-sarcoglycan gene for correction of limb-girdle muscular dystrophy reported a detectable AAV capsid-specific T cell response in 3 patients. In one patient this response came up very rapidly on day 2 after gene transfer and may have contributed to his loss of AAV-transduced muscle cells (92). Additional studies are needed to further determine the risk induction of T cells upon intramuscular injection of AAV vectors. It will be especially important to further elucidate the role of regulatory T cells in preventing immune-mediated destruction of AAV-transduced muscle cells.

AAV vectors are directly infused into the central nervous system to treat neurological diseases such as Alzheimer (93), Parkinson’s disease (94–97), infantile neuronal ceroid lipofuscinosis (98), Canavan disease, a N-acetylaspartate storage disease of the brain caused by mutations of the aspartoacylase gene (99) and others. Studies did not assess AAV capsid or transgene product-specific T cell responses following AAV gene transfer. Some trials analyzed serum antibody responses to AAV and reported that they increased in some patients suggesting that enough vectors had leaked from the injection sites to trigger a peripheral B cell response.

Considering that the central nervous system is an immunoprivileged site that lacks the lymphatic structures needed for activation of immune responses it is unlikely that enough vector will leak into the periphery to activate AAV capsid or transgene-product-specific CD8^+^ T cells which could then cross the blood brain barriers and attack vector-transduced cells. Nevertheless, it would be prudent to monitor patients for AAV-induced T cell responses.

Intracoronary application of an AAV vector expressing SERCA2a to modulate calcium metabolism reported transient increases in AAV capsid-specific T cell frequencies in 1 out of 9 patients without any clinical consequences or further changes in blood chemistry (100). Intravenous application of an AAV9 vector expressing the survival motor neuron 1 gene for treatment of spinal muscular atrophy reported increases in liver enzymes in some of their patients (101). As T cell responses were not analyzed, the etiology of this observation remains uncertain.

As one would expect, AAV vectors induce adaptive immune responses especially if given at high doses to peripheral sites. It remains unclear how common T cells to AAV capsid or the encoded transgene product interfere with sustained therapeutic benefits as many trials still fail to test for T cell responses. Injections of even large doses of AAV does not inevitably lead to detectable T cell responses with the obvious caveat that T cell assays have sensitivity limits and may not reveal small responses. Furthermore, the activity of tissue resident memory CD8^+^ T cells may not be spotted by monitoring increases in circulating AAV capsid- or transgene product-specific T. cells. It will be important for gene therapist that base their therapeutics on AAV to further define factors that promote CD8^+^ T cell responses. Vector dose and route of administration clearly play roles. CpG content within the vector genome may affect responses as was shown in mice (98). The capsid itself may have an effect. Age can have an effect; younger people tend to mount better immune responses, but older people are more likely to have immunological memory to AAVs. The underlying disease especially if it causes local inflammatory reaction can affect treatment outcome. The HLA type of the gene therapy recipient will affect if and how many epitopes of the capsid can be recognized by T cells. The type of the transgene and its similarity to endogenous proteins will determine if the host is tolerant or responsive.

After the initial AAV gene transfer trials for hemophilia B indicated that AAV-induced CD8^+^ T cells may cause loss of cells producing F.IX (8), one of the next trials very carefully monitored serum F.IX and transaminase levels following AAV gene transfer. The two patients that received the highest vector dose showed by weeks 7 or 9 modest subclinical increases in aspartate aminotransferase and alanine aminotransferase and decreases in F.IX. They were treated for 9 or 4 weeks, respectively with prednisolone, which reduced transaminase levels and stopped further declines in F.IX (5). Steroids, such as prednisolone, are in general well tolerated if given for a short period of time. They are widely used in transplantation medicine, to treat auto-immune diseases or conditions caused by overwhelming inflammatory responses. They affect multiple aspects of T cell responses resulting in reduced cytokine production and decreased proliferation. Specifically, they dampen T cell activation by reducing phosphorylation of key signaling molecules of the T cell receptor (102). They induced production of anti-inflammatory cytokines and they promote proliferation of regulatory T cells (103). Although steroids are not suitable as a monotherapy to prevent transplant rejection they have been used successfully by Nathwani (5) and others (79, 104, 105) to prevent loss of AAV-transduced cells upon hepatic gene transfer. Nevertheless, in other trials steroids did not avert gradual loss of the transgene product (106–108). The optimal regimen for steroid therapy remains to be established (109). While most trials carefully monitored serum enzyme levels and started steroid treatment once transaminases increased others gave steroids to all patients immediately or shortly after treatment. The former approach is not only cumbersome as it requires weekly testing but also carries the risk that treatment could be initiated too late. The latter approach has the disadvantage that patients, who would not mount immune-mediated rejection are treated unnecessarily. It also remains unclear how long steroids should be given and if this time frame depends on the type of vector or differs for each patient. As steroids have not always prevented loss of transgene-expressing cells additional immunosuppressives need to be explored. In a trial with an AAV1 vector for correction of lipoprotein lipase deficiency, a 12 week course of methylprednisolone together with cyclosporin and mycophenolate mofetil was started shortly before AAV transfer; the drugs did not prevent increases in AAV capsid-specific T cells, but T cells appeared to be functionally impaired and failed to achieve removal of vector-transduced muscle cells (110). Cyclosporin inhibits T cell activation by blocking signaling through NFAT transcription factors, which are regulators of CD8^+^ T cell functions (111) as well as the NF-κB and activator protein 1 (AP-1) pathways. Cyclosporin is also being explored in pre-clinical models for dampening of AAV capsid-specific neutralizing antibody responses (112). Nevertheless, cyclosporin depending of timing of antigen exposure *versus* drug treatment was shown in a cardiac allograft rat model to prevent activation of regulatory T cells (113), while in other systems cyclosporin augmented the activity of this immunosuppressive cell subset (112, 113). Mycophenolate mofetil inhibits inosine monophosphate dehydrogenase, the rate-limiting enzyme for synthesis of guanosine nucleotides which are essential for cell cycle progression of proliferating lymphocytes (114).

Other drugs that are being considered are TLR antagonists or drugs that target downstream molecules of PRR signaling to inhibit activation of the inflammatory responses that are essential to drive activation of naïve T cells (115). Such drugs could block numerous steps of PRR activation pathways such as TIRAP, MyD88, IKK-γ, NF-κB, or IRAK-4. Inflammatory responses could also be dampened by blocking TNF-α signaling through monoclonal antibodies such as Remicade, Humira, Cimzia, the receptor fusion protein Enbre or the small molecule inhibitor pentoxifylline (116). Drugs are also available to block type I IFN signaling by inhibiting IRF-3 or JAK1 which would affect T cell maturation (117). All of these drugs would impair primary T cell responses but may not block recall responses. Induction of CD4^+^ T cells could be inhibited by preventing antigen presentation through the MHC class II pathway through chloroquine, which prevents acidification of endosomes (118), cyclosporin A and tacrolimus, which both inhibit calcineurin (119) or blockers of the lysosomal protease cathepsin S, such as morpholinurea-leucine-homophenylalanine-vinylsulfone-phenyl (120). Some of the drugs such as cyclosporin A and tacrolimus also block MHC class I antigen presentation, which can also be inhibited by proteasome inhibitors such as lactacystin (121) or inhibitors of TAP (122). T cell activation is inhibited by some of the drugs that are already being used such as steroids, cyclosporin A and mycophenolate mofetil. Others could be tried. These include belatacept, a fusion protein of CTLA-4 and the Fc portion of IgG-1, which binds CD80 and CD86 and thereby blocks CD28 signaling (123). Dapirolizumab pegol is an antibody against CD40L, which blocks another co-stimulatory pathway. This drug is currently undergoing phase III trials for treatment of systemic lupus erythematosus  (124, 125). Rapamycin was tested preclinically and was shown to block humoral and cellular immune responses to AAV and to increase induction of regulatory CD4^+^ T cells (126–128). Rapamycin is an mTOR inhibitor that blocks cell cycle progression. When used during T cell activation it prevents generation of effector CD8^+^ T cells but promotes memory formation (129).

Immunosuppression although potentially essential for some patients to ensure therapeutic benefits of AAV-mediate gene transfer comes at a cost. Immunosuppressive drugs have side effects that are unrelated to inhibition of immune responses. To name a few, prednisolone can cause intestinal ulcers, tacrolimus can result in headaches and muscle pain, belatacept has been linked to intestinal problems as has mycophenolate mofetil. Immunosuppression by its very nature robs an individual of its ability to fight off pathogens thus heightening the risks of more serious infections associated with more severe disease and increased shedding of the pathogen.

## Summary

The immune system evolved to respond to components of pathogens including those of viral vectors. This system, which is essential for survival of an organism, is built on redundancy to counter rapidly mutating pathogens that have come up with multiple ways to dodge immune-mediated destruction. Modifying AAV vectors by removing parts that induce inflammatory responses or provide epitopes for T cell recognition may at best blunt responses. Nevertheless, modern medicine has developed a multitude of drugs that prevent activation of the immune system to overcome rejection of organ transplants. This in turn provides blueprints for drugs that can effectively block destructive T cell responses. Organs have been transplanted successfully since 1954 while AAV was not discovered until the mid-1960s. It then took another 30 years before an AAV vector was tried in a human gene therapy trial. By 2008, AAV-mediated gene therapy reported clinical benefits for a congenital blindness and then by 2011 systemic AAV transfer to the liver showed clinical benefits for hemophilia B patients. AAV-mediated gene transfer has thus progressed from its infancy to a stage of adolescence where hopefully remaining problems such as immune-mediated rejection can be solved in the near future.

## Author Contributions

The author confirms being the sole contributor of this work and has approved it for publication.

## Funding

Wistar Institute.

## Conflict of Interest

The author has in the past been the recipient of a Sponsored Research Agreement by Spark Therapeutics.

The author declares that the research was conducted in the absence of any commercial or financial relationships that could be construed as a potential conflict of interest.
